# Liquid biopsies for the diagnosis of early-stage gastric cancer: A 5-year systematic review

**DOI:** 10.1016/j.jlb.2026.100465

**Published:** 2026-04-08

**Authors:** Efthymia Papaioannou, Despoina Ioannou, Theodora Papamitsou, Matthaios Bobos, Michalis Aivaliotis, Konstantina Psatha, Evangelos Karamitrousis

**Affiliations:** a Histologistas Research Group, Interinstitutional Postgraduate Program Health and Environmental Factors, Medical School, Faculty of Health Sciences, Aristotle University of Thessaloniki, 54124, Thessaloniki, Greece; b Laboratory of Histology-Embryology, Medical School, Faculty of Health Sciences, Aristotle University of Thessaloniki, 54124, Thessaloniki, Greece; c Department of Biomedical Sciences, School of Health Sciences, International Hellenic University, 57400, Thessaloniki, Greece; d Laboratory of Biochemistry, School of Medicine, Faculty of Health Sciences, Aristotle University of Thessaloniki, GR-54124, Thessaloniki, Greece

**Keywords:** Gastric cancer, Oncology, Biomarkers, Liquid biopsy

## Abstract

Gastric cancer remains one of the leading causes of cancer-related mortality worldwide, largely due to its late-stage diagnosis. Liquid biopsy has emerged as a promising, minimally invasive method for early cancer detection, leveraging circulating biomarkers such as nucleic acids, extracellular vesicles, and tumor cells.

**Objective:**

This systematic review aimed to evaluate the emerging role of liquid biopsy as a diagnostic tool for the early detection of primary gastric cancer, focusing on the past five years of published research.

**Methods:**

Following PRISMA guidelines and based on the PICO framework, a comprehensive literature search was conducted across PubMed and Scopus databases, yielding 620 articles. After screening and eligibility assessment, 16 studies were included. Quality evaluation was performed using the QUADAS-2 tool and Analytical Validation Summaries.

**Results:**

The included studies demonstrated consistently high diagnostic performance of various liquid biopsy-derived biomarkers. Notably, circulating non-coding RNAs-particularly miRNAs, circRNAs, lncRNAs, and tsRNAs-showed high sensitivity and specificity in early-stage gastric cancer detection. DNA methylation signatures, cfDNA fragmentomics, lipidomic profiles, and folate receptor-positive CTCs, also emerged as valuable diagnostic modalities. Most studies reported area under the curve (AUC) values exceeding 0.85, with several outperforming conventional serum markers, such as CEA and CA19-9.

**Conclusions:**

Liquid biopsy holds significant promise as a non-invasive, accurate diagnostic approach for early gastric cancer. RNA-based and cfDNA-based biomarkers, in particular, exhibit strong potential for integration into routine screening protocols. Further large-scale, prospective validation studies are warranted to support clinical translation and standardization.

## Highlights

• Liquid biopsy enables accurate, non-invasive early detection of gastric cancer
• Non-coding RNAs show strong diagnostic performance in early-stage disease
• CfDNA methylation, fragmentomics and omics offer complementary biomarkers
• Multi-analyte liquid biopsy panels outperform conventional serum markers
• Curated cohorts limit real-world screening applicability and clinical translation

## Graphical abstract

**Figure:**
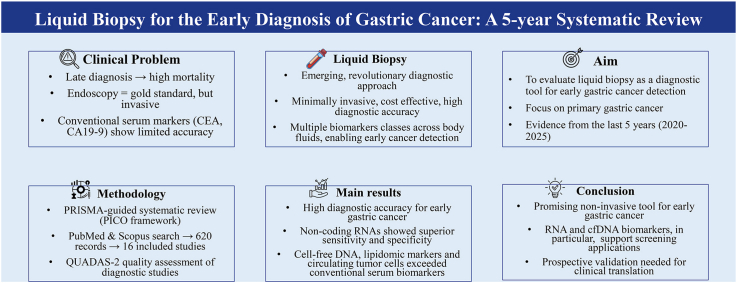

## 1 Introduction

Gastric cancer constitutes one of the most common malignancies [1] and ranks as the fifth most diagnosed malignancy worldwide, with over one million new cases annually [2]. It is more commonly diagnosed in male patients of Asian or African-American descent, with a median age of onset at 70 years and peak incidence observed in the 75–84 age group [3]. As the third leading cause of cancer-related mortality [2], and considering its inherently complex and heterogeneous nature, coupled with the fact that the majority of patients remain asymptomatic until the disease reaches an advanced stage [4], the urgent need for novel and effective strategies to enable early and timely diagnosis, is underscored.

Currently, upper gastrointestinal (UGI) series and endoscopic evaluation represent the cornerstone modalities in clinical practice for the detection of early-stage gastric cancer, serving as the established diagnostic gold standard [5]. In this context, liquid biopsy techniques have recently emerged and are attracting growing interest as a promising non-invasive alternative [5]. However, given the limited sensitivity and specificity of conventional serum-based biomarkers, recent research has increasingly focused on more stable and informative molecular targets identified not only in serum, but also in alternative biofluids such as gastric juice and peritoneal lavage [6]. This systematic review aims to explore recent advancements in the application of liquid biopsy techniques for the early detection of primary gastric cancer. Given the rapid technological evolution of liquid biopsy approaches—particularly with the integration of high-throughput omics technologies and next-generation sequencing—this review focuses on studies published within the last five years (2020–2025), in order to provide a contemporary and methodologically consistent overview of current diagnostic developments.

### 1.1 Definition, classification and risk factors of gastric cancer

Gastric cancer (GC), deriving from the mucosal epithelium of the stomach, is a highly lethal malignancy, with locally advanced gastric cancer associated with a five-year survival rate of nearly 35%, declines sharply to about 7% in the context of metastatic progression, whereas early-stage detection is associated with markedly improved outcomes, reaching survival rates of up to 95-99% [7]. Histologically, over 90% of cases are adenocarcinomas, which the World Health Organization (WHO) classifies into tubular, papillary, mucinous, poorly cohesive, and mixed subtypes. The Lauren classification further distinguishes intestinal, diffuse, and mixed types, while anatomically, tumors are categorized as proximal or distal [7]. On a molecular level, The Cancer Genome Atlas (TCGA) defines four distinct subtypes based on genomic alterations: chromosomal instability (CIN), microsatellite instability (MSI), Epstein–Barr virus (EBV)-positive, and genomically stable (GS) tumors [8].

GC arises from a complex interplay of infectious, environmental, and genetic factors. *Helicobacter pylori*, a helical Gram-negative bacterium residing in the gastric mucosa, has been associated with an elevated risk of developing GC [40], while chronic gastric inflammation, tobacco use, excessive alcohol intake, obesity, and consumption of salt-preserved or processed meat, further elevate the risk. Occupational exposure to asbestos, metals, and high-temperature particulates, as well as Epstein–Barr virus (EBV) infection, prior gastric surgery or radiation, and dietary carcinogens like nitrosamines, have also been implicated. Additionally, inherited mutations and demographic factors such as male sex, low socioeconomic status, and certain racial/ethnic groups contribute to increased susceptibility [7,9].

### 1.2 Definition and advantages of liquid biopsy

Liquid biopsy is a minimally invasive approach that analyzes biological fluids to detect tumor-derived components, released into circulation during cellular apoptosis or necrosis, thereby providing valuable molecular information regarding tumor presence, burden, and progression [10]. While tissue biopsy remains the gold standard for establishing diagnosis, performing histopathological classification, and evaluating biomarkers, liquid biopsy has emerged as a low-risk, and cost-effective alternative. Its clinical value is particularly evident in metastatic disease, in patients with anatomically inaccessible tumors, unfit patients for invasive procedures, and in the early detection of subclinical malignancies. Furthermore, its capacity to capture both intra- and inter-tumoral heterogeneity, facilitate serial sampling for real-time disease monitoring, and identify minimal residual disease (MRD), highlights its growing relevance in precision oncology [10,11].

### 1.3 Biomarkers

Classical serum biomarkers such as carcinoembryonic antigen (CEA), cancer antigen 19-9 (CA19-9), and CA125 are commonly used in GC for monitoring purposes. However, their diagnostic performance remains limited. Reported positivity rates in early gastric cancer are particularly low, approximately 4.3% for CEA, 4.8% for CA19-9, and 1.9% for CA125, reflecting their poor sensitivity for early detection ([12]; Haque et al., 2022). Other protein biomarkers—including CA72-4, pepsinogen I/II ratio, trefoil factor 3 (TFF3), and alpha-fetoprotein (AFP)—have shown potential for improved risk stratification, detection of recurrence, or identification of aggressive tumor phenotypes, yet most remain inadequately validated for routine clinical application and show poor sensitivity [5].

In addition to protein-based markers, another promising biomarker is circulating tumor cells (CTCs), which are malignant cells that detach from the primary tumor and enter the peripheral bloodstream—often via epithelial–mesenchymal transition—facilitating metastatic spread. Their presence, quantity, and molecular characteristics have been associated with disease progression, prognosis, and response to therapy [13]. Moreover, circulating cell-free deoxy-ribo-nucleic acid (cfDNA), and more specifically, circulating tumor DNA (ctDNA), consists of nucleic acid fragments released into the bloodstream by tumor cells through apoptosis or necrosis, offering real-time molecular insights due to their short half-life. ctDNA harbors tumor-specific genetic and epigenetic alterations [14], which refer to reversible changes in the structure of nucleic acids and histone proteins within DNA molecules [15], including mutation and methylation patterns, which show promise for early GC detection—particularly through methylation-based assays—yet technical limitations in sensitivity, stability, and tumor specificity remain significant barriers to routine clinical application [14]. Moreover, the detection of cfDNA could pave the way for improved insights into disease prognosis and facilitate the evaluation of response to specific therapeutic interventions [16].

Expanding beyond DNA, circulating cell-free ribo-nucleic acid (cfRNA) molecules—including microRNAs (miRNAs), long non-coding RNAs (lncRNAs), circular RNAs (circRNAs), piwi-interacting RNAs (piRNAs), and messenger RNAs (mRNAs)—have emerged as promising liquid biopsy biomarkers in GC [14]. Among these, circulating miRNAs are the most extensively studied. miRNAs are small non-coding RNAs that regulate gene expression post-transcriptionally by binding to the 3′-prime untranslated region (UTR) of mRNAs, thereby inhibiting translation and they play key roles in cancer development, acting as oncogenes or tumor suppressors, and their expression profiles can classify tumor types [17]. Their expression signatures can reflect tumor subtype and biological behavior, while their high stability in circulation, combined with notable diagnostic sensitivity and specificity, supports their potential as non-invasive biomarkers for early detection and prognostication. Nonetheless, their clinical utility remains under investigation due to methodological inconsistencies and limited validation in large-scale studies [14].

Similarly, among circulating cell-free RNAs, lncRNAs—transcripts exceeding 200 nucleotides without protein-coding potential—have gained significant attention in GC due to their regulatory roles in transcription, epigenetics, and tumor progression. Multiple lncRNAs have been identified as dysregulated in GC and linked to diagnostic and prognostic value. SNHG17, HULC, ZNFX1-AS1, B3GALT5-AS1, HOXC-AS3, HOST2, XIST, FTX, DDX11-AS1, LINC01234, ABHD11-AS1, ARAP1-AS1, LUCAT1, CRNDE, and BCAR4 are among those most studied. These lncRNAs have been associated with increased cell proliferation, invasion, metastasis, progression, apoptosis inhibition, drug resistance, and poor overall or disease-free survival. Although lncRNAs demonstrate considerable potential as biomarkers for gastric cancer, their clinical translation remains limited by incomplete mechanistic understanding and significant technical challenges, related to their low abundance, instability, and susceptibility to degradation [18].

Furthermore, circRNAs constitute a structurally unique subclass of non-coding RNAs, characterized by covalently closed-loop structures that lack 5′ caps and 3′ polyadenylated tails, rendering them highly stable, resistant to exonucleases, such as RNase R, and associated with prolonged half-life and preservation in body fluids. Recent advances in high-throughput sequencing have accelerated the identification of circRNAs involved in GC pathogenesis, with several candidates emerging as potential biomarkers [19]. Additionally, piRNAs are small, evolutionarily conserved non-coding RNAs (24–31 nt) that interact with PIWI proteins to form silencing complexes involved in transposon repression, genome stability, and epigenetic regulation [20]. With over 20,000 human piRNAs identified, they significantly outnumber microRNAs and are increasingly linked to tumorigenesis, as their dysregulation correlates with cancer progression and distinct clinicopathological features [20]. Among the 8759 piRNAs identified in gastric tissue, 50 are differentially expressed between GC patients and healthy controls, with piRNA-1245, piR-651, and piR-823 showing strong associations with tumor size, stage, metastasis, and prognosis, while their involvement in the regulation of numerous protein-coding genes, suggests a functional role in gastric tumorigenesis and supports their utility as promising non-invasive biomarkers for diagnosis and prognosis [21].

Also, mRNAs have recently emerged as promising non-invasive biomarkers in multiple cancers, including GC. However, the clinical use of cf-mRNAs for the early diagnosis of GC is still constrained by several challenges, including their fragmented nature, low concentration, limited stability, and the presence of contaminating cellular RNA [14]. In addition, transfer RNA-derived small RNAs (tsRNAs) are a distinct class of non-coding RNAs generated through specific cleavage of mature or precursor tRNAs. Rather than random degradation products, they are functionally active molecules involved in gene regulation. Recent studies suggest that dysregulated tsRNA expression may serve as a promising biomarker for the early detection and prognosis of GC [22].

Beyond nucleic acids, exosomes represent another key avenue of liquid biopsy research. Exosomes are nanosized extracellular vesicles derived from the endocytic pathway, typically measuring 30 to 150 nm, and are present in numerous body fluids [23]. Their lipid bilayer structure protects various bioactive molecules, including RNAs such as miRNAs and lncRNAs from degradation, enabling them to mediate intercellular communication in GC. Specific exosomal miRNAs, like miR-29s, found to be downregulated in patients with peritoneal metastases, are linked to poor prognosis, while elevated levels of exosomal lncRNA myocardial infarction-associated transcript (MIAT), correlate with reduced survival and disease relapse, highlighting their potential utility as minimally invasive biomarkers for GC diagnosis, prognosis, and monitoring through liquid biopsy [24].

Lastly, emerging studies have started to investigate the significance of serum lipid-related indicators as potential biomarkers in GC. Metabolic syndrome components—such as insulin resistance, abnormal lipid profiles, and impaired glucose metabolism—have been associated with increased cancer susceptibility and tumor progression. Notably, disruptions in serum lipid levels appear to be linked to GC development. Biomarkers like the triglyceride-glucose (TyG) index and the atherosclerotic index (AI) have demonstrated diagnostic value in other diseases, though their specific role in GC prognosis and early detection, remains insufficiently defined [25].

### 1.4 Aim of the study

The primary objective of this systematic review is to evaluate the diagnostic performance and clinical utility of emerging liquid biopsy biomarkers - including non-coding RNAs, cfDNA methylation patterns, and circulating tumor cells - for the detection of primary early-stage gastric cancer, as well as their ability to distinguish malignant disease from healthy, benign, or precancerous gastric conditions when such comparator groups were available. By synthesizing evidence from the preceding five years according to PRISMA guidelines and the PICO framework, this study aims to identify high-performance molecular signatures that could complement or serve as non-invasive alternatives to traditional endoscopic screening. This objective guides the methodological flow of the review, moving from a structured literature search to a qualitative synthesis of biomarker efficacy and a critical appraisal of their potential for clinical translation.

## 2 Materials and methods

### 2.1 PICO model

The research question for this systematic review was clearly defined using the PICO framework (Table 1). The identification, screening, and evaluation of articles followed the PRISMA (Preferred Reporting Items for Systematic Reviews and Meta-Analyses) guidelines. The completed PRISMA 2020 checklist is provided as Supplementary File S3, while the PRISMA-S checklist (focused on the search strategy) is available as Supplementary Table S1. Quality assessment tables were tailored to align with the specific study designs included. This review was not prospectively registered in PROSPERO or another public registry. Nevertheless, in order to assess potential overlap with ongoing or registered reviews in this area, a structured search of the PROSPERO database was performed using combinations of terms related to gastric cancer, liquid biopsy, and early detection; the findings of this search are presented in the Critical View section. The lack of prospective registration is acknowledged as a methodological limitation. To enhance transparency, all methodological steps—including the review objective, eligibility criteria, search strategy, study selection process, and quality assessment—are explicitly reported in accordance with PRISMA 2020 principles.

**Table 1:** PICO table outlining Population (P), Intervention (I), Comparator (C), and Outcome (O).

PICO	
Population (P)	Human subjects evaluated for the detection of early-stage primary gastric cancer, including patients with early-stage gastric cancer and comparator groups such as healthy controls, benign gastric disease, precancerous gastric lesions, and, where applicable, other malignancies used for specificity assessment.
Intervention (I)	Use of liquid biopsy for early diagnosis
Comparator (C)	Traditional tissue biopsy, standard imaging modalities
Outcome (O)	Findings indicated several biomarkers that detect early stomach/gastric cancer

### 2.2 PRISMA flow diagram

**Fig. 1:**
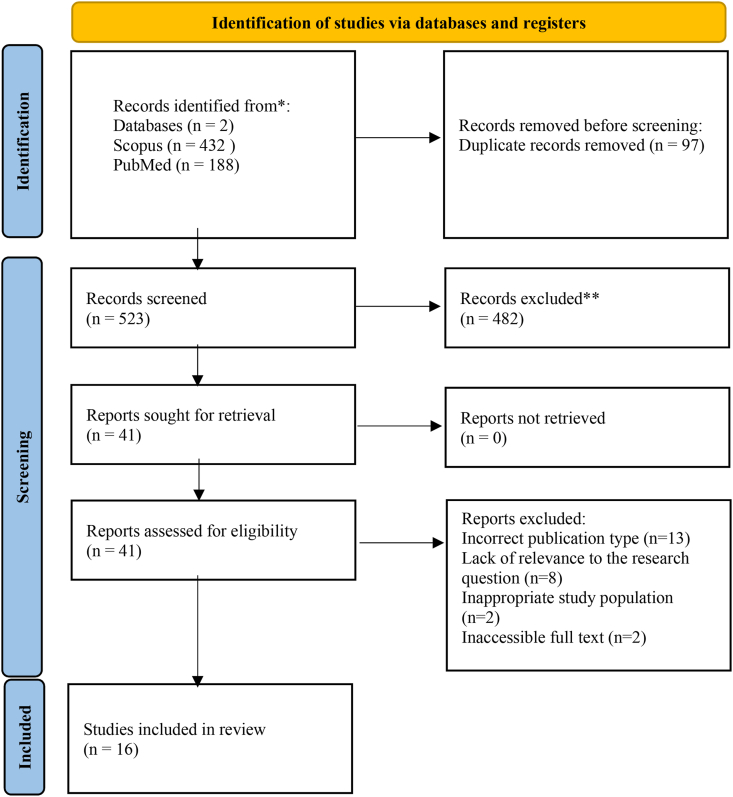
Prisma flow diagram.

A PRISMA flow diagram (Fig. 1) was created to depict the process of study selection. The search strategy combined free-text keywords, truncation, and Boolean operators as follows: (“Stomach cancer” OR “Stomach neoplasm∗” OR “Gastric cancer” OR “Gastric neoplasm∗” OR “Gastric oncology” OR “Stomach oncology”) AND (“liquid biops∗” OR “biomarker testing” OR “non-surgical biops∗”). The inclusion criteria covered publications from 2020 to 2025, with the last search conducted in June 2025. The full search strategies for each database are provided in Supplementary Table S2. The search strategy was developed and internally reviewed by the author team to ensure methodological consistency and transparency. Eligible studies included primary research articles (e.g., observational and diagnostic studies) that evaluated liquid biopsy-based biomarkers for the diagnosis of early-stage primary gastric cancer in human subjects. Comparator populations, where included, could consist of healthy controls, patients with benign gastric disease, or individuals with precancerous gastric lesions. In some studies, additional comparator groups included patients with other malignancies, primarily for specificity assessment. Studies focusing exclusively on metastatic disease, prognosis, treatment response, or non-gastric malignancies were excluded. To be included, studies had to be published in English. Articles were accessed through available open-access sources and institutional access where possible. No studies were excluded solely on the basis of access limitations. Only research articles were included. Although systematic reviews and meta-analyses were excluded from the final qualitative synthesis to prioritize primary diagnostic data, these records were analytically utilized for manual reference tracking. The bibliographic lists of all identified reviews were systematically screened (manual reference screening) to ensure the inclusion of any relevant primary studies not captured by the initial electronic search. This technique was employed to minimize the risk of missing eligible studies and to strengthen the overall evidence base of the review. The literature search was conducted using PubMed (NCBI) to access MEDLINE records, Scopus (Elsevier) databases through their native database interfaces. Grey literature sources were not included in the search strategy. Two independent reviewers performed the screening of all articles. Any disagreements were resolved through discussion and consensus; if consensus could not be reached, a third reviewer was consulted to make the final decision. An initial record of the total number of retrieved articles was made before applying the inclusion criteria. The screening process evaluated the relevance of studies based on their titles, abstracts, and full texts. Duplicate records were manually identified and removed before screening. No automation tools were employed. Manual reference checking of eligible articles was conducted to identify any additional relevant studies. No contact with authors for unpublished data was undertaken. A detailed table was compiled to list all studies that met the inclusion criteria for this systematic review (Table 2). Extracted data included study design, study aim, methodological approach, population characteristics, investigated biomarkers, and main diagnostic findings, including reported performance metrics where available.

**Table 2:** Summary of the results from the 16 selected studies.

#	Study	Type of study	Aim	Method	Population	Biomarkers	Main Results
1	Roy et al. [27]	Observational study	To identify and validate a panel of circRNA biomarkers, developing a noninvasive liquid biopsy assay with high specificity and improved performance over traditional tumor markers for the early detection of gastric cancer.	Candidate circRNAs identified via limma analysis of GSE89143 & GSE83521; validated by RT-qPCR in 28 matched GC/normal tissues. Liquid biopsy developed using serum from 2 retrospective cohorts (92/46 & 102/48 GC/controls) and 1 prospective cohort (n = 24 pre-/post-surgery). Specificity tested in colorectal, esophageal, pancreatic, and hepatocellular cancers (n = 20 each). Receiver operated characteristics (ROC), AUC, logistic regression, Kruskal-Wallis, paired *t*-test (p < 0.05).	28 pairs GC and adjacent normal mucosa (ANM) tissues for pilot validation, serum samples from two retrospective GC cohorts (92 GC patients and 46 controls; 102 GC patients and 48 controls), 24 prospectively enrolled GC patients with pre- and post-surgery serum samples, and 20 patients for each non-GC gastrointestinal cancer type [colorectal cancer (CRC), esophaheal squamous cell carcinoma (ESCC), pancreatic ductal adenocarcinoma (PDAC), hepatocellular carcinoma (HCC)] for specificity evaluation.	hsa_circ_0045602, hsa_circ_0008768, hsa_circ_0007380, hsa_circ_0002019, hsa_circ_0006089, hsa_circ_0034398, hsa_circ_0052001, and hsa_circ_0001013	An 8-circRNA serum panel was developed for non-invasive GC detection (AUC = 0.87 training, 0.83 validation), with strong early-stage performance. Biomarkers outperformed classic markers and showed high specificity vs other GI cancers. Post-operatively decreases confirmed tumor specificity.
2	Tang et al. [28]	Retrospective study	To discover novel non-invasive biomarkers for the early diagnosis of GC.	Exosomal miRNAs were extracted from serum samples of early-stage GC patients and healthy controls using ExoQuick and miRNeasy kits. Small RNA libraries were prepared and sequenced (Illumina HiSeq2000), followed by bioinformatics analysis (DESeq, miREvo, mirdeep2) for differential expression. Selected miRNAs were validated by RT-qPCR, and diagnostic performance was assessed using ROC curves, logistic regression, and fold-change analysis (p < 0.05).	36 early-stage GC patients, 12 age- and gender-matched healthy controls, and a validation cohort of 50 newly recruited early-stage GC patients with 50 matched healthy individuals.	Serum exosomal miR-92b-3p, −146b-5p, -9-5p, and let-7g-5p.	Serum exosomal miR-92b-3p, miR-146b-5p, miR-9-5p, and let-7g-5p demonstrated superior diagnostic performance compared to conventional tumor markers. Their combined application achieved the highest diagnostic accuracy, highlighting their potential as a non-invasive biomarker panel for early detection of GC.
3	Kamkar et al. [17]	Computational bioinformatics analysis	To develop a non-invasive, liquid-biopsy based assay by using circRNAs as molecular biomarkers for early detection of GC.	Weighted miRNA co-expression network analysis was applied to 972 serum miRNA profiles from 13 cancer types and healthy controls to identify key miRNAs based on module membership and trait significance. Preservation analysis and machine learning were used to develop and evaluate two panels: one for multi-cancer detection and another specific to GC.	Serum samples from 13 different cancer types and non-cancer controls. GC detection specifically: Train set: 40 GC + 100 healthy controls, Test set: 50 GC + 1541 other (12 cancer types + controls), Validation set: Early-stage GC patients (from GSE164174).	4 miRNAs: miR-1228-5p, miR-1343-3p, miR-6765-5p, miR-6787-5p.	The 4-miRNA serum panel (miR-1228-5p, miR-1343-3p, miR-6765-5p, miR-6787-5p) achieved high diagnostic performance (AUC = 86.87%, accuracy: 87%, sensitivity: 89%, specificity: 90%). External validation confirmed strong specificity (88.33%), and the panel effectively distinguished GC from other gastrointestinal cancers.
4	Izumi et al. [29]	Retrospective study	To evaluate the use of circulating microRNAs as non-invasive diagnostic biomarkers for GC.	Four-phase design: (1) Biomarker discovery using TCGA miRNA-seq data; (2) Tissue validation via qRT-PCR on 50 paired samples (Kumamoto Univ.); (3) Retrospective serum validation (547 samples, Kumamoto and Nagoya); (4) Prospective serum validation (349 participants, Jiangsu Univ.). A 3-miRNA signature was developed using logistic regression with elastic net. Analysis conducted using R (limma, glmnet, pROC).	>1900 tissue and serum samples from patients with gastric cancer, adjacent normal tissue, and healthy controls across Japan and China.	miR-18a, miR-181b, and miR-335.	A 3-miRNA circulating signature (miR-18a, miR-181b, miR-335) was developed through multi-phase biomarker discovery and validated in retrospective and prospective cohorts. It demonstrated strong diagnostic performance (AUC up to 0.86), outperformed conventional markers, remained effective for early-stage GC, and was specific to GC. Risk scores increased across disease stages, and cost-effectiveness modeling favored this assay over endoscopy, indicating its clinical utility in population screening.
5	Kahroba et al. [30]	Observational study	To identify GC-specific miRNAs within serum-derived exosomes and to evaluate their potential as non-invasive diagnostic biomarkers.	Serum exosomes were isolated and characterized by dynamics light scattering (DLS), transmission electron microscopy (TEM(, and Western blotting for CD9, CD81, and CD63. Total RNA was extracted and screened using a focused miRNA PCR panel, with four miRNAs (miR-10a-5p, miR-19b-3p, miR-215-5p, miR-18a-5p) validated via stem-loop RT-qPCR. Statistical analysis included ROC curves, Mann-Whitney *U* test, Analysis of variance (ANOVA), and Spearman correlation.	43 paired primary gastric adenocarcinoma tissue samples and adjacent non-malignant tissue samples were collected. Peripheral blood was obtained from the same patients and 40 healthy controls.	miR-10a-5p, miR-19b-3p, miR-215-5p, and miR-18a-5p.	miR-10a-5p, miR-19b-3p, miR-215-5p, and miR-18a-5p were significantly upregulated in both GC tissues and serum-derived exosomes, with individual AUCs ranging from 0.72 to 0.82 and combined signatures achieving high diagnostic accuracy (AUC = 0.865 in tissues, 0.813 in exosomes). A significant positive correlation was observed between tumoral and exosomal miRNA expression, strongest for miR-10a-5p. Furthermore, miRNA expression levels correlated with tumor size, lymph node involvement, and distant metastasis, but not with patient age, gender, or histological subtype.
6	Yu et al. [31]	Diagnostic case-control study	To evaluate whether exosomal miRNAs derived from neutrophils (Neu-Exo miRNAs) can serve as accurate and non-invasive biomarkers for the diagnosis of GC, and to develop an efficient method for their isolation and detection from human serum samples.	Neu-Exo were isolated from neutrophil cultures and serum using ultra-centrifugation and CD66b-coupled Dynabeads. Neu-Exo were characterized by TEM, nanoparticle tracking analysis (NTA), and western blotting, and quantified via dual antibody-based flow cytometry. RNA was extracted for miRNA sequencing, with candidate miRNAs validated by qRT-PCR and droplet digital PCR (ddPCR).	For miRNA quantification using qRT-PCR, the study analyzed 61 GC patients, 36 benign gastric disease (BGD) patients, and 49 healthy controls. For further diagnostic validation using ddPCR, 52 GC patients, 31 BGD patients, and 34 healthy controls were evaluated. Additionally, RNA sequencing was performed on Neu-Exo samples from 3 GC patients and 3 healthy donors, while diagnostic performance of CD66b^+^ Neu-Exo was validated in a separate cohort of 27 GC patients and 27 healthy controls.	CD66b^+^ Neu-Exo, miR-223-3p and miR-425-5p.	The study demonstrated that CD66b^+^ Neu-Exo and their cargo miRNAs, particularly miR-223-3p and, to a lesser extent, miR-425-5p, are promising non-invasive biomarkers for GC detection. ddPCR analysis of miR-223-3p in Neu-Exo showed excellent diagnostic accuracy, with AUCs exceeding 0.9 across key clinical comparisons, including early-stage disease. These findings highlight the clinical potential of Neu-Exo and their miRNA content in liquid biopsy strategies for the early and accurate diagnosis of GC.
7	Gao et al. [32]	Research article	To develop a highly sensitive and specific dual-signal amplification platform by integrating catalytic hairpin assembly (CHA) with clustered regularly interspaced short palindromic repeats (CRISPR)Cas12a and a self-replicating CHA system (SRCHA), in order to enable the non-invasive and accurate detection of miR-182 and CEA in serum samples, and to construct a logic-based diagnostic model for staging GC.	A dual-signal detection system based on CRISPR-Cas12a and CHA/SRCHA was developed to detect serum miR-182 and CEA in gastric cancer. Fluorescence assays were used to measure Cas12a-mediated cleavage for miR-182 and N-methyl mesoporphyrin IX (NMM)-based signal generation for CEA. Native polyacrylamide gel electrophoresis (PAGE) verified nucleic acid structural changes, and biomarker levels were assessed in patient and control serum samples.	30 individuals: 10 healthy donors, 10 patients with early-stage gastric cancer (stage I–II), and 10 patients with advanced-stage disease (stage III–IV).	CEA, miR-182	Gao et al. [26] developed a highly sensitive dual-amplification platform combining CHA, CRISPR-Cas12a, and SRCHA for non-invasive detection of GC biomarkers. The system detected circulating miR-182 [limit of detection (LOD): 0.063 fM] and CEA (LOD: 4.8 pg/mL) with high specificity and stability, accurately identifying GC and distinguishing early from advanced stages, highlighting its potential for liquid biopsy–based early diagnosis.
8	Guo et al. [33]	Observational diagnostic study	To develop and validate a non-invasive, blood-based diagnostic tool using extracellular vesicle -derived lncRNAs, aiming to detect early-stage gastric cancer with high accuracy and specificity	This three-phase diagnostic study identified GClnc1 as a candidate lncRNA biomarker for early GC, validated its expression in plasma extracellular vesicles via qRT-PCR across multicenter cohorts, and confirmed its stability, specificity, and post-operative decline. Performance was assessed using ROC analysis, sensitivity/specificity estimates, and multivariable models, including comparisons with other gastrointestinal (GI) cancers (HCC, PDAC, CRC).	A total of 2141 participants were included across three phases: 888 patients with GC (including both early-stage and advanced-stage), 158 patients with chronic atrophic gastritis, 193 with intestinal metaplasia, 501 healthy donors, and 401 patients with other GI cancers (colorectal, pancreatic, and hepatocellular).	Extracellular vesicle-derived lncRNA GClnc1.	This study identified and validated the extracellular vesicle-derived lncRNA GClnc1 as a robust, non-invasive biomarker for the early detection of GC. It showed excellent diagnostic accuracy across multiple cohorts, outperformed traditional markers, and distinguished early GC from precancerous lesions and biomarker-negative patients. GClnc1 levels decreased after tumor resection, remained stable in clinical samples, and were specific to GC, supporting its potential role in transforming early screening and improving patient outcomes through liquid biopsy.
9	Yuanet al. [33]	Observational diagnostic study	This study investigates the potential of transfer tsRNAs as biomarkers for the diagnosis of GC, particularly in its early stages.	Total RNA from serum, tissues, and GC cell lines was analyzed via small RNA sequencing and differential expression using EdgeR, with tsRNA identification based on Mitochondrial and Nuclear tRNA fragment database (MINTbase/MINTmap). Selected tsRNAs were validated by qRT-PCR and Sanger sequencing. Bioinformatic analyses included target prediction, pathway enrichment, and structural annotation, while diagnostic performance was assessed via ROC/AUC and statistical tests using t-tests, logistic regression, and Cox models.	60 patients with pathologically confirmed gastric cancer, 60 healthy controls (serum samples), 36 paired gastric tissue samples (tumor and adjacent non-tumor tissues)	tsRNAs, specifically: Upregulated: tRF-31-PNR8YP9LON4VD, tRF-30-MIF91SS2P4FI, tRF-30-IK9NJ4S2I7L7 Downregulated: tRF-38-W6RM7KYUPRENRHD2, tRF-37-LBRY73W0K5KKOV2, tRF-36-JB59V3WD8YQ84VD, tRF-25-MBQ4NKKQBR, tRF-36-0KFMNKYUHRF867D.	High-throughput sequencing identified 435 dysregulated tsRNAs in gastric cancer, with three upregulated and five downregulated tsRNAs validated by qRT-PCR. The upregulated tsRNAs showed strong diagnostic performance (AUCs >0.84 in early-stage GC) and correlated with tumor, nodes, metastases (TNM) stage and tumor depth; combined, they achieved an AUC of 0.956, outperforming CEA and CA199 as non-invasive biomarkers.
10	Cai et al. [34]	Observational diagnostic study	To develop and validate a serum lipid metabolic signature (SLMS) as a non-invasive biomarker for the early diagnosis and prognosis prediction of gastric cancer using machine learning algorithms and lipidomics profiling.	Fasting serum samples underwent lipid extraction and Ultra-Performance Liquid Chromatography-Tandem Mass Spectrometry (UPLC-MS/MS) analysis for untargeted and targeted lipidomics, with lipid identification via MS-Data Independent Analysis (DIAL). Feature selection used Partial Least Squares Discriminant Analysis (PLS-DA) and correlation-based filtering, with Lipid data analyzer (LDA) applied for diagnosis and Generalized linear model (Glmnet) for subtype prediction. Model performance was assessed via 10-fold cross-validation and ROC analysis, while spatial metabolomics and transcriptomics of GC tissues supported metabolic subtype characterization.	944 participants including GC patients (early and advanced stages), individuals with precancerous gastric lesions, and healthy donors, with 266 GC patients and 266 healthy donors forming the main exploration cohort.	Serum lipidomic profile—specifically a panel of 19 lipids forming the Serum Lipid Metabolic Signature (SLMS).	This study developed a SLMS using machine learning on lipidomics data from GC patients and healthy donors. The SLMS accurately distinguished GC cases (AUC up to 0.993) and outperformed traditional markers, especially in early-stage and biomarker-negative patients. It also identified prognostic subtypes (GCPS) with distinct survival outcomes. Multi-omics analyses confirmed widespread lipid metabolism disturbances in GC, supporting the clinical value of SLMS for diagnosis and prognosis.
11	Hideura et al. [35]	Observational diagnostic study	The study aimed to establish and assess the diagnostic performance of a highly sensitive Combined restriction digital PCR (CORD) assay for detecting RUNX3 methylation in serum DNA, with the goal of enabling early, non-invasive detection of GC using minimal sample volumes.	Serum samples from EGC patients and healthy controls were collected and processed for DNA extraction. Methylated RUNX3 was detected using the CORD assay, which combines methylation-sensitive enzyme digestion with ddPCR. Statistical analyses included ROC curves, logistic regression, and various univariate tests to assess diagnostic performance and clinical correlations.	50 patients with EGC and 61 control individuals.	Methylated RUNX3.	The CORD assay detected methylated RUNX3 in EGC with 50% sensitivity and 80.3% specificity, outperforming traditional serum markers like CEA and CA19-9. Elevated methylated RUNX3 levels were significantly associated with tumor size, lymphovascular invasion, and deep submucosal infiltration. Post-treatment levels decreased significantly, supporting its potential as a non-invasive biomarker for early detection and treatment monitoring.
12	Nakamura et al. [36]	Observational diagnostic study	To develop and validate a diagnostic index that combines demographic factors (age and sex) with molecular biomarkers (human telomerase reverse transcriptase [hTERT] expression and methylated RUNX3) to improve the detection of EGC.	In this retrospective study, serum samples from 94 EGC patients and 225 healthy controls were analyzed using the CORD assay to quantify methylated RUNX3 and hTERT levels. DNA was extracted from 0.4 mL of serum and analyzed via ddPCR, while CEA, CA19-9, and anti-*H. pylori* antibodies were measured using commercial kits. Statistical analyses included Mann-Whitney *U* test, χ^2^ test, ROC analysis, and multivariate logistic regression.	319 serum samples collected prior to treatment from 94 patients with EGC (limited to the submucosa) and 225 healthy controls	Methylated RUNX3 and hTERT copy number.	The Age, sex, hTERT and methylated RUNX3 (ASTEm-R3) index, developed using the CORD assay, demonstrated superior diagnostic performance for EGC compared to conventional markers (CEA, CA19-9) and offers a non-invasive alternative to endoscopy. It shows potential for screening high-risk individuals, such as those with *H. pylori* infection or atrophic gastritis. Further validation in prospective and non-Japanese cohorts is needed, as current findings are based on pre-treatment serum samples.
13	Yu et al. [41]	Observational study	The development of a non-invasive, cfDNA-based liquid biopsy assay for the sensitive detection of EGC.	Plasma cfDNA was collected pre-diagnosis and sequenced at 5x coverage. Four cfDNA features were analyzed: fragment size, copy number variations (CNVs), nucleosome positioning, and single nucleotide variants (SNV) signatures. A two-layer ensemble model (elastic-net, random forest, XGBoost, neural networks) was trained and optimized via cross-validation, showing high diagnostic accuracy. Simulated screening in a 100,000-person cohort compared performance with gastroscopy. Statistical analysis used R (Wilcoxon, Fisher's exact, Jonckheere tests; p < 0.05).	Α prospective cohort of 110 patients with stage I–II GC and 139 non-cancer individuals, a first validation cohort of 73 GC patients and 94 non-cancer individuals, and a second independent validation cohort of 47 GC patients and 49 non-cancer individuals.	Μulti-dimensional cfDNA features, including fragment size patterns, CNVs, nucleosome coverage patterns (NCPs), and single nucleotide substitution (SNS) signatures, derived from low-coverage whole-genome sequencing.	The study showed that cfDNA profiling can accurately distinguish GC from non-cancer cases, including early-stage disease, using a machine learning model with high sensitivity and specificity. This noninvasive method outperformed gastroscopy in simulated population screening, supporting its potential for early GC detection.
14	Seo et al. [37]	Observational diagnostic study	To investigate whether characteristics of methylated ctDNA in plasma can reflect those of GC tissue and to evaluate their diagnostic utility for GC using fragmentomics and 5′ end-motif analysis.	Plasma and tissue samples from 22 GC patients and plasma from 40 healthy donors were analyzed using methylated DNA immunoprecipitation sequencing (MeDIP-seq) to assess cfDNA methylation and fragmentation patterns. Bioinformatic processing included alignment to hg38 and identification of differentially methylated regions (DMRs) and co-methylated regions (CMRs). Cancer patients showed distinct methylation profiles, higher short fragment ratios (SFR), and enriched T-end 5′ motifs. ROC analysis showed high diagnostic performance, with AUC values up to 0.95 for CpG islands.	40 Healthy Donors, 22 GC patients.	Methylated cfDNA, with emphasis on (DMRs), SFR, and 5′ end motifs as features of tumor-derived cfDNA	Over 1.5 million DMRs were identified, mostly hypermethylated in cancer, with approximately 41% overlap between tissue and plasma. GC patients showed significantly higher SFRs, especially in CpG islands (AUC = 0.95), and distinct 5′ end motifs, with enrichment of T-end motifs in cancer. These findings underscore the diagnostic potential of cfDNA methylation and fragmentomics for non-invasive detection of GC.
15	Qi et al. [38]	Observational diagnostic study	To identify and validate GC–specific methylation biomarkers derived from plasma cfDNA, in order to develop a non-invasive, highly sensitive and specific model for early detection of GC.	This prospective case-control study analyzed cfDNA from 150 gastric cancer patients and 100 controls using cfMeDIP-seq to identify DMRs. A least absolute shrinkage and selection operator(LASSO)-selected 21-marker panel was used to train a random forest model with high diagnostic accuracy, validated in internal and external datasets. Propensity score matching and TCGA data supported the robustness of the findings.	100 non-tumor controls and 150 GC patients.	DMRs in cfDNA.	The study identified 1663 predominantly hypomethylated cfDNA regions in GC patients, with elevated cfDNA levels particularly in carcinoma in situ and cardia tumors. Using LASSO regression, 21 key methylation markers were selected, and a random forest model based on these achieved high diagnostic accuracy (AUC = 0.9877), independent of stage, age, sex, or cfDNA concentration, with performance further enhanced by including cfDNA levels. External validation, supported the robustness and clinical potential of these markers.
16	Cao et al. [39]	Observational diagnostic study	To investigate whether folate receptor α (FRα)-positive CTCs could be used as a noninvasive liquid biopsy approach in GC.	FR^+^ CTCs were isolated from blood using ligand-targeted PCR and quantified in FU/3 mL. Folate receptor 1 (FOLR1) expression was assessed via immunofluorescence, and TCGA data supported bioinformatics analysis. Statistical tests included t-tests and ROC analysis (p < 0.05).	143 GC patients (30 early-stage, 113 advanced) and 20 with BGD who underwent FR^+^ CTC testing.	FRα-positive CTCs, FR^+^ CTCs.	The study highlighted the overexpression of FRα in GC cells and its association with poor prognosis. FR^+^ CTCs were significantly elevated in GC patients, especially in those with vascular or perineural invasion, regardless of overall stage. FR^+^ CTC detection outperformed traditional markers like CEA and emerged as a promising non-invasive diagnostic tool for early detection and monitoring of GC.

### 2.3 Methodological quality assessment

To evaluate the quality and risk of bias in diagnostic accuracy studies, the QUADAS-2 (Quality Assessment of Diagnostic Accuracy Studies 2) tool was utilized, independently by two reviewers. This tool assesses four key domains—patient selection, index test, reference standard, and flow and timing—providing a structured framework to determine the risk of bias and applicability concerns for each study. Each domain is rated as having low, high, or unclear risk of bias. Additionally, for studies involving diagnostic test development or validation, an Analytical Validation Summary was compiled, focusing on key parameters such as sensitivity, specificity, precision, accuracy, -LOD, and reproducibility. This summary enabled a standardized comparison of methodological rigor and technical performance across different studies.

## 3 Results

### 3.1 PRISMA flow diagram

A total of 620 records were initially identified through database searching (PubMed = 188; Scopus = 432). After the removal of duplicate records (n = 97), 523 records remained for title and abstract screening. Following the application of eligibility criteria—including publication date (2020–2025), language (English), study design (primary research articles), and relevance to the research question—482 records were excluded as ineligible. A total of 41 articles were assessed for full-text eligibility. Of these, 25 articles were excluded for the following reasons: incorrect publication type (n = 13), inaccessible full text (n = 2), inappropriate study population (n = 2), and lack of relevance to the research question (n = 8), including studies not focused on gastric cancer or focusing on metastatic disease.

A total of 16 studies were ultimately included in the qualitative synthesis and are summarized in Table 2.

### 3.2 Methodological quality assessment

Additionally, three different quality assessments were prepared, including the Quality Assessment of Diagnostic Accuracy Studies 2 (QUADAS-2) and the Analytical Validation Summary.

#### 3.2.1 QUADAS-2

The QUADAS-2 tool was used to assess the quality and validity of the included diagnostic accuracy studies. All 16 studies included in the qualitative synthesis were systematically evaluated across four key domains: patient selection, index test, reference standard, and flow and timing. Most studies, such as those by Roy S. et al., and Tang S. et al., demonstrated a consistently low risk of bias in patient selection, index test, reference standard, and flow and timing domains, with minimal applicability concerns. However, a few studies exhibited areas of uncertainty or concern; for example, Kamkar L. et al. showed unclear risk in patient selection, index test, and reference standard, combined with high risk in flow and timing, as well as high applicability concerns across patient selection and index test domains. Similarly, Seo SY et al. was rated high risk for patient selection and moderate risk in index test and reference standard, reflecting potential limitations in study design or reporting. Several studies, including Yu D. et al. and Yu P. et al.[41], contained unclear or moderate risks across multiple domains, highlighting variability in methodological quality. Overall, the majority of studies maintained low risk and good applicability, supporting the reliability of their diagnostic findings, while a subset required cautious interpretation due to identified biases (Table 3).

**Table 3:** Application of QUADAS-2 for 16 studies.

#	Study	Risk of bias				Applicability Concerns		
		Patient Selection	Index Test	Reference Standard	Flow and Timing	Patient Selection	Index Test	Reference Standard
1	Roy S et al. [27]	Low	Low	Low	Low	Low	Low	Low
2	Tang S et al. [28]	Low	Low	Low	Low	Low	Low	Low
3	Kamkar L et al. [17]	Unclear	Unclear	Unclear	High	High	High	Moderate
4	Izumi D et al. [29]	High	High	Low	Unclear	Moderate	Moderate	Low
5	Kahroba H et al. [30]	High	High	Low	Unclear	Moderate	Moderate	Low
6	Yu D et al. [31]	Unclear	Unclear	Low	Unclear	Unclear	Moderate	Low
7	Gao R et al. [26]	Unclear	Low	Low	Low	Unclear	Low	Low
8	Guo X et al. [32]	Low	Low	Low	Low	Low	Low	Low
9	Yuan J et al. [33]	Low	Low	Low	Low	Low	Low	Low
10	Cai Z-R et al. [34]	Low	Low	Low	Low	Low	Low	Low
11	Hideura E et al. [35]	Low	Low	Low	Low	Low	Low	Low
12	Nakamura K et al. [36]	Low	Moderate	Low	Low	Low	Low	Low
13	Yu P et al. [41]	Unclear	Unclear	Low	Unclear	Unclear	Moderate	Low
14	Seo SY et al. [37]	High	Moderate	Moderate	Low	Moderate	Low	Low
15	Qi J et al. [38]	Low	Low	Moderate	Low	Low	Low	Low
16	Cao B et al. [39]	Low	Low	Low	Low	Low	Low	Low

## 4 Discussion

Based on the systematic review and the data collected in accordance with the PRISMA guidelines and PICO model, the following conclusions can be derived from the overview provided in Table 2. Among the sixteen studies included in the final synthesis, a consistent trend emerged regarding the promising diagnostic utility of liquid biopsy in the early detection of gastric cancer and several investigations reported high diagnostic accuracy for specific circulating biomarkers.

More specifically, nine of the included studies highlighted a growing trend toward the utilization of circulating RNA species—particularly circRNAs, miRNAs, lncRNAs, and tsRNAs—as promising non-invasive biomarkers. circRNAs were investigated in the study by Roy et al. [27]; miRNAs were explored in the studies by Tang et al. [28], Kamkar et al. [17], Izumi et al. [29], Kahroba et al. [30], Yu, D. et al. [31], and Gao et al. [26]; lncRNAs were evaluated in the study by Guo et al. [32]; and tsRNAs were assessed in the study by Yuan et al. [33].

In particular, Roy et al. [27] identified a panel of eight circRNAs (hsa_circ_0045602, hsa_circ_0008768, hsa_circ_0007380, hsa_circ_0002019, hsa_circ_0006089, hsa_circ_0034398, hsa_circ_0052001, and hsa_circ_0001013) that demonstrated strong diagnostic accuracy for early-stage GC (AUC = 0.87 in the training cohort and 0.83 in the validation cohort). The panel exhibited excellent specificity when compared to other GI malignancies and showed a marked decrease in expression post-surgery, supporting its tumor specificity and clinical utility in early detection.

Beyond circRNAs, a substantial body of evidence within the reviewed literature, also supports the diagnostic potential of miRNAs in the context of liquid biopsy for early GC detection, with six of the included studies identifying miRNAs as key biomarkers. In particular, the study by Tang et al. [28] investigated four serum exosomal miRNAs—miR-92b-3p, miR-146b-5p, miR-9-5p, and let-7g-5p—which demonstrated superior diagnostic performance, compared to conventional tumor markers such as CEA. Notably, the combined use of these miRNAs, yielded the highest diagnostic accuracy, supporting their value as a non-invasive biomarker panel for early-stage GC.

In a complementary approach, Kamkar et al. [17] applied network biology and machine learning techniques to develop a four-miRNA panels (miR-1228-5p, miR-1343-3p, miR-6765-5p, and miR-6787-5p) that achieved high diagnostic accuracy (AUC = 86.87%) and effectively distinguished GC from other GI malignancies. Further supporting the role of miRNAs in early detection, Izumi et al. [29] developed a three-miRNA circulating signature (miR-18a, miR-181b, and miR-335), which demonstrated robust diagnostic accuracy (AUC up to 0.86), outperformed traditional markers (CEA and CA19-9), and remained effective across early disease stages.

Kahroba et al. [30] also demonstrated that exosomal miR-10a-5p, miR-19b-3p, miR-215-5p, and miR-18a-5p were significantly upregulated in GC patients, with combined signatures achieving high diagnostic accuracy (AUC = 0.813 for exosomes). Notably, their expression levels correlated with tumor size and metastasis, reinforcing their relevance as non-invasive biomarkers. In line with these findings, Yu, D. et al. [31] investigated Neu-Exo as a novel source of circulating biomarkers, identifying miR-223-3p and miR-425-5p as significantly upregulated in GC patients. Using a CD66b-based isolation system and ddPCR, miR-223-3p achieved excellent diagnostic accuracy across all comparisons (AUC up to 0.930), underscoring the potential of Neu-Exo-derived miRNAs as highly specific and non-invasive tools for early GC detection.

In alignment with the above evidence, the study by Gao et al. [26] introduced an innovative CRISPR-Cas12a–assisted dual-signal amplification platform that enabled ultrasensitive detection of circulating miR-182 and CEA in serum, achieving exceptional diagnostic accuracy for both early and advanced GC. Complementing the miRNA-focused approaches, the study by Guo et al. [32] highlighted the diagnostic potential of lncRNAs and identified GClnc1 as a promising biomarker for EGC. EV-derived GClnc1 achieved excellent diagnostic performance (AUC = 0.9369), outperforming conventional markers (CEA, CA72-4, CA19-9), and remained accurate across disease stages underscoring its utility as a stable, cancer-specific, and non-invasive tool for early detection.

Expanding the landscape of non-coding RNA biomarkers, the study by Yuan et al. [33] investigated tsRNAs and validated eight differentially expressed tsRNAs, three were upregulated (tRF-31-PNR8YP9LON4VD, tRF-30-MIF91SS2P4FI, and tRF-30-IK9NJ4S2I7L7) and five were downregulated (tRF-38-W6RM7KYUPRENRHD2, tRF-37-LBRY73W0K5KKOV2, tRF-36-JB59V3WD8YQ84VD, tRF-25-MBQ4NKKQBR, and tRF-36-0KFMNKYUHRF867D) in tumor tissues and serum of GC patients. The three upregulated tsRNAs not only correlated with tumor burden, early TNM stage, and other clinicopathological parameters, but also demonstrated strong diagnostic accuracy (AUCs: 0.808–0.843), which was further enhanced when combined (AUC = 0.956). Importantly, their diagnostic performance in early-stage GC (TNM I/II) surpassed that of conventional serum tumor markers such as CEA, CA19-9, and CA72-4 (AUCs: 0.564–0.578), highlighting their value as sensitive and non-invasive biomarkers for early detection and risk stratification.

These findings contribute to the growing recognition of non-coding RNAs as promising and biologically relevant biomarkers for liquid biopsy applications. However, their potential clinical integration requires further validation in well-designed prospective studies, as well as direct comparative evaluation against established diagnostic approaches. Notably, several RNA signatures—such as the circRNA panel proposed by Roy et al. [27], the EV-derived lncRNA GClnc1 in Guo et al. [32], the three-miRNA signature by Izumi et al. [29], and the tsRNA combination by Yuan et al. [33]— outperformed traditional biomarkers in sensitivity, specificity, and AUC values, especially in early-stage GC. These findings collectively support the growing recognition of non-coding RNAs as robust, stable, and cancer-specific tools for liquid biopsy applications, and highlight their potential role in complementing or even replacing existing markers in population-level screening and early diagnosis efforts.

Building on the exploration of non-coding RNAs, the study by Cai et al. [34] introduced an innovative lipidomic approach to liquid biopsy for GC and proposed a machine learning-derived SLMS composed of 19 lipids, which achieved high diagnostic accuracy (especially in early-stage GC) and outperformed conventional markers (CEA, CA19-9, CA72-4). Additionally, the SLMS showed potential in detecting precancerous lesions, while a derived prognostic model (GCPS) effectively stratified patients by survival outcomes. These results highlight the value of lipid-based liquid biopsy as a promising complementary tool for early detection and prognosis in GC.

In addition to RNA-based biomarkers, several studies investigated epigenetic alterations in cfDNA, particularly DNA methylation, as a promising approach for early GC detection through liquid biopsy. Among these, both Hideura et al. [35] and Nakamura et al. [36] focused on RUNX3 promoter methylation, with the latter combining it with hTERT copy number in the ASTEm-R3 index. Expanding beyond single-gene targets, Yu, P. et al. [31] developed a cfDNA-based assay that integrates multiple cfDNA characteristics, while Seo et al. [37] and Qi et al. [38] emphasized the diagnostic value of DMRs, SFRs, and 5′ end motifs.

Further exploring methylation-based biomarkers, Hideura et al. [35] assessed serum methylated RUNX3 using the bisulfite-free CORD assay, which achieved a sensitivity of 50% for early GC—markedly higher than that of conventional methylation assays (0–19%) and significantly superior to traditional serum markers such as CEA and CA19-9. Notably, elevated levels of methylated RUNX3 were significantly associated with tumor size, lymphovascular invasion, and deep submucosal infiltration, highlighting its potential clinical utility for both early detection and guiding treatment decisions.

Building upon these findings, Nakamura et al. [36] introduced the ASTEm-R3 index, which combined methylated RUNX3 and hTERT copy number using the CORD assay. This index outperformed conventional markers (CEA, CA19-9) and offered a non-invasive alternative to UGI endoscopy for EGC detection, with potential application in screening high-risk individuals.

Extending the investigation of cfDNA-based diagnostics beyond methylation, Yu, P. et al. [31] developed a comprehensive liquid biopsy assay that integrates multiple cfDNA features—fragment coverage, CNV, transcription start site (TSS) coverage, and mutational signatures. This multi-dimensional approach achieved exceptional diagnostic performance across multiple cohorts (AUROC = 0.937–0.972), with sensitivity up to 94.1% and specificity consistently above 90%. Notably, the model accurately identified early-stage GC across tumor locations and clinical subtypes, outperforming gastroscopy in large-scale simulations and underscoring its potential as a powerful, non-invasive alternative for early GC screening.

Continuing this line of research, Seo et al. [37] combined methylation and fragmentation-based analyses of cfDNA to distinguish GC patients from healthy individuals. The study identified over 1.5 million DMRs, with a predominance of hypermethylation in cancer cases. In addition, a significantly higher SFR in CpG islands and distinct 5′ end-motif patterns were observed in cancer patients and the integration of these features yielded strong diagnostic performance (AUC = 0.95), underscoring the promise of cfDNA profiling for early, non-invasive GC detection.

Following this trajectory, Qi et al. [38] further reinforced the value of cfDNA methylation profiling by analyzing plasma samples from GC patients and controls, identifying 1663 DMRs—primarily hypomethylated—in tumor samples. From these, 21 key markers were selected using LASSO regression and used to construct a random forest diagnostic model, which demonstrated excellent performance (AUC = 0.9877), consistent across all disease stages and clinical subgroups. The inclusion of cfDNA concentration, further improved accuracy, and external validation confirmed that the methylation and expression profiles of the selected genes could reliably distinguish GC from normal tissue, highlighting their strong potential for early, stage-independent detection.

Collectively, the studies investigating cfDNA-based biomarkers consistently demonstrated high diagnostic performance, often exceeding that of traditional serum markers, although these comparisons were not uniformly performed within the same study populations. Both Hideura et al. [35] and Nakamura et al. [36] showed that the detection of methylated RUNX3, particularly when incorporated into the ASTEm-R3 index, outperformed conventional markers and offered a viable non-invasive alternative to endoscopy. Similarly, the comprehensive cfDNA profiling approach by Yu, P. et al. [31] achieved AUROC values up to 0.972 and outperformed gastroscopy in simulation models, highlighting its clinical potential for early detection. Seo et al. [37] and Qi et al. [38] further validated the utility of DMR-based and fragmentation-integrated cfDNA analysis, achieving AUCs of 0.95 and 0.9877 respectively. These findings underscore the promise of cfDNA methylation-based strategies as highly sensitive, specific, and non-invasive diagnostic tools that could complement existing diagnostic approaches in population-level GC screening.

Beyond nucleic acid-based biomarkers, Cao et al. [39] highlighted the diagnostic potential of FR^+^ CTCs as an emerging liquid biopsy tool for GC. In this cohort, FR^+^ CTC levels were significantly elevated compared to benign controls and showed superior diagnostic performance for early-stage disease (AUC = 0.736) relative to serum CEA (AUC = 0.593), achieving a sensitivity of 80%. Levels of FR^+^ CTCs, also correlated with vascular and perineural invasion and tended to increase with advancing pathologic stage. Notably, FRα-positive tumor cells were detected in para-tumoral blood vessels, reinforcing the potential of FRα as a molecular tag for identifying CTCs and enhancing early detection through liquid biopsy.

### 4.1 Liquid biopsy in precancerous lesions and secondary prevention

Beyond early-stage adenocarcinoma, several analyzed studies demonstrate the potential of liquid biopsy to identify high-risk precancerous states, which is critical for effective secondary prevention. For instance, the GClnc1 lncRNA biomarker successfully distinguished early gastric cancer (EGC) from precancerous lesions, including chronic atrophic gastritis and intestinal metaplasia. Similarly, the Serum Lipid Metabolic Signature (SLMS) proved capable of detecting precancerous gastric lesions, suggesting that metabolic reprogramming occurs prior to malignant transformation. From a screening perspective, the ASTEm-R3 index - which incorporates methylated *RUNX3* - offers a non-invasive alternative to endoscopy for monitoring high-risk individuals, such as those with persistent *H. pylori* infection or atrophic gastritis. These findings suggest that multi-analyte liquid biopsy panels could be integrated into secondary prevention protocols to identify patients requiring more intensive endoscopic surveillance.

## 5 Conclusion

In conclusion, liquid biopsy demonstrates strong potential as a non-invasive, accurate approach for the early detection of GC. Non-coding RNAs (including circRNAs, miRNAs, lncRNAs, and tsRNAs) demonstrate consistently high diagnostic performance in early gastric cancer detection. Several studies reported improved sensitivity and specificity compared to conventional tumor markers; however, these findings should be interpreted with caution due to study heterogeneity, differences in study design, and the limited number of direct head-to-head comparisons within the same patient cohorts. These RNA-based biomarkers offer stable, cancer-specific profiles that remain effective across early disease stages. Similarly, cfDNA analysis, particularly through methylation profiling and fragmentation-based techniques, has emerged as a powerful tool, providing high accuracy and the ability to detect cancer at early, often asymptomatic stages. Complementary approaches, including lipidomic profiling and detection of CTCs, further enhance the diagnostic landscape. Collectively, these advancements highlight the evolving role of liquid biopsy in GC screening. By enabling earlier diagnosis through minimally invasive methods, these biomarkers offer promising pathways for improving patient outcomes and reducing the burden of late-stage disease. As research continues to refine and validate these approaches, their integration into clinical practice could transform current diagnostic strategies. Specifically, by identifying high-risk precancerous lesions, liquid biopsy could serve as a foundational tool for secondary prevention, allowing for targeted intervention before the onset of invasive adenocarcinoma. This would support more effective, population-wide screening efforts and significantly improve long-term patient outcomes.

## 6 Critical view section

This systematic review examined the use of liquid biopsy over the last five years (2020–2025) with the specific aim of evaluating its role in the early detection of primary gastric cancer. Studies focusing on metastatic disease, prognosis, or treatment monitoring were deliberately excluded. Accordingly, the review provides a diagnostic, translation-oriented synthesis of recent biomarker classes-including circulating and exosomal RNAs, cfDNA methylation and fragmentomics, lipidomic signatures, and FRα-positive circulating tumor cells-rather than a comprehensive overview of all liquid biopsy applications in gastric cancer. The depth of coverage is best described as a structured qualitative synthesis with methodological appraisal, and no meta-analysis was performed due to substantial heterogeneity among included studies with respect to index tests, reference standards, study populations, and experimental methodologies.

Different viewpoints coexist within this research field and are reflected in the included studies. A technology-forward perspective emphasizes the frequently high diagnostic accuracy reported and the promise of multi-analyte panels as potential enhancements to conventional serum biomarkers. A clinically pragmatic viewpoint is more conservative, noting that many studies rely on case-control designs comparing gastric cancer patients with healthy individuals, which may not adequately reflect real-world screening conditions. In such settings, the principal challenge lies in distinguishing early gastric cancer from precancerous lesions, benign gastric diseases, and inflammatory conditions under variable disease prevalence. A third, implementation-focused viewpoint acknowledges the strong diagnostic promise of liquid biopsy—particularly RNA- and cfDNA-based biomarkers—while underscoring the practical barriers to clinical adoption. A primary challenge in secondary prevention remains the ability of these assays to reliably differentiate between early malignancy and benign inflammatory conditions or low-grade dysplastic lesions in a real-world screening population.

From our perspective, a defining feature of the current literature is the absence of a universal biomarker for early gastric cancer, with the field instead converging toward two complementary strategies: circulating RNA-based panels, especially circular and other non-coding RNAs demonstrating consistently high diagnostic performance with scalable molecular workflows, and cfDNA-based approaches-including methylation, fragmentomics, and multi-feature models-that may better capture tumor-derived signals when single targets are insufficient. This convergence is reinforced by the rapid emergence of omics technologies, which allow multidimensional characterization of tumor biology through liquid biopsy. Nevertheless, a major limitation-and, to some extent, an inherent paradox-of the existing evidence is the discrepancy between excellent diagnostic performance reported in curated cohorts and the limited proof of clinical benefit in population-level screening. This gap, together with substantial heterogeneity in analytical and pre-analytical protocols and the ongoing lack of adequately powered, high-quality multicenter prospective trials, continues to hinder clinical implementation. Importantly, the limited number of studies performing direct comparisons between liquid biopsy biomarkers and conventional serum markers within the same patient populations further restricts the ability to draw definitive conclusions regarding their relative diagnostic performance. Furthermore, a potential limitation of this study is the inclusion of exclusively papers published in English language and the primary reliance on the PubMed and Scopus databases. Although these databases offer comprehensive coverage of the most significant peer-reviewed literature in the field, this strategy may have excluded specific records indexed exclusively in other databases such as Embase or Cochrane CENTRAL, or early-stage data presented solely in conference proceedings. Future updates to this synthesis should consider a multi-database approach to further minimize the risk of publication bias. Beyond methodological considerations, these challenges are further amplified by pronounced inter- and intra-tumor heterogeneity and by differences in diagnostic and therapeutic strategies between Eastern and Western healthcare systems. We therefore argue that future research should prioritize standardized laboratory workflows, independent prospective validation in high-risk populations, and head-to-head comparisons with endoscopy-based diagnostic pathways, supported by transparent reporting of pre-analytical variables, formal assessments of clinical utility, and strengthened cross-disciplinary and international collaboration.

Finally, this review distinguishes itself from much of the existing and ongoing registered work by maintaining a narrow diagnostic focus on early detection and by integrating evidence across multiple biomarker modalities. A comprehensive search of PROSPERO identified several ongoing systematic reviews and meta-analyses that predominantly emphasize cfDNA/ctDNA-based approaches, prognostic or treatment-response applications, machine learning models, or cancer types beyond gastric cancer. In contrast, the present synthesis adopts a diagnostic-oriented perspective centered on early gastric cancer and incorporates non-coding RNA panels, cfDNA methylation and fragmentomics, lipidomic signatures, and CTC-based assays. This cross-modality approach offers a more holistic view of the evolving liquid biopsy landscape, helping to clarify where evidence is more mature and where it remains limited, thereby supporting more rigorous future study design and facilitating clinically meaningful translation toward early gastric cancer screening.

## Definitions of sex and/or gender

Sex and gender were not considered analytical parameters in this systematic review, as the study focused on the identification and evaluation of diagnostic biomarkers within the context of liquid biopsy applications for the early detection of gastric cancer. Any sex- or gender-related information, when available, was retrieved exclusively from the original studies and reported as presented therein.

## Author contributions

Conceptualization: E.*P. and* E.K.; Data curation: E.P., D.I., and E.K.; Formal analysis: E.P., D.I., and E.K.; Funding acquisition: E.K.; Investigation: E.P., D.I., and E.K.; Methodology: E.P., D.I., and E.K.; Project administration: E.*P. and* E.K.; Resources: E.P., D.I., and E.K.; Software: E.P.; Supervision: E.P., T.P., and E.K.; Validation: E.P., T.P., and E.K.; Visualization: E.P.; Writing – original draft: E.P., D.I., and E.K.; Writing – review and editing: E.P., D.I., T.P., and E.K.

## Data availability statement

The original contributions presented in this study are included in the article. Further inquiries can be directed to the corresponding authors.

## Ethical approval-patient consent

No ethical approvals or patient consent were necessary for the study.

## Declaration of generative AI and AI-assisted technologies in the manuscript preparation process

During the preparation of this work, the authors used OpenAI to improve language, clarity, and readability. After using this tool, the authors reviewed, edited, and verified the content as needed and take full responsibility for the content of the published article.

## Funding

No external funding was received for this study.

## Conflicts of interest

The authors declare that there are no conflicts of interest.
