# Supplementary material for: Liquid biopsies for the diagnosis of early-stage gastric cancer: A 5-year systematic review
Source: J Liq Biopsy. 2026 Apr 8;12:100465. doi: 10.1016/j.jlb.2026.100465 (PMC13092028; doi:10.1016/j.jlb.2026.100465)
Supplement: Multimedia component 1 [file mmc1.docx]

**Supplementary Table S1. PRISMA-S checklist**

| **Item** | **Checklist Item** | **Location in Manuscript** | **Reported?** |
| --- | --- | --- | --- |
| 1 | Database name(s) | *Materials and Methods → PRISMA Flow Diagram (“PubMed, Scopus”)* | Yes |
| 2 | Database provider/platform | *Materials and Methods → PRISMA Flow Diagram (NCBI, Elsevier)* | Yes |
| 3 | Search interface used | *Materials and Methods → PRISMA Flow Diagram (“native database interfaces”)* | Yes |
| 4 | Date of last search | *Materials and Methods → PRISMA Flow Diagram (“last search conducted in June 2025”)* | Yes |
| 5 | Date range covered | *Materials and Methods → PRISMA Flow Diagram (“Publications from 2020 to 2025”)* | Yes |
| 6 | Full search strategy for each database | *Materials and Methods → Supplementary table S2* | Yes |
| 7 | Use of Boolean operators, truncation, MeSH/subject headings | *Materials and Methods → PRISMA Flow Diagram and Supplementary table S2* | Yes |
| 8 | Use of filters/limits (e.g., humans, language) | *Materials and Methods → PRISMA Flow Diagram and Supplementary table S2* | Yes |
| 9 | Grey literature sources searched | *Materials and Methods → PRISMA Flow Diagram (“Grey literature sources were not included in the search strategy.”)* | Yes |
| 10 | Manual reference checking | *Materials and Methods → PRISMA Flow Diagram (“Manual reference checking of eligible articles was conducted to identify any additional relevant studies. ”)* | Yes |
| 11 | Contact with authors for unpublished data | *Materials and Methods → PRISMA Flow Diagram (“No contact with authors for unpublished data was undertaken .”)* | Yes |
| 12 | Language restrictions applied | *Supplementary table S2* | Yes |
| 13 | Search strategy peer-reviewed | *Materials and Methods → PRISMA Flow Diagram (“The search strategy was developed and internally reviewed by the author team to ensure methodological consistency and transparency”)* | Yes |
| 14 | Deduplication method | *Materials and Methods → PRISMA Flow Diagram (“Duplicate records were manually identified and removed before screening*.*”)* | Yes |
| 15 | Automation tools used in screening | *Materials and Methods → PRISMA Flow Diagram (“Νo automation tools were employed”)* | Yes |
| 16 | Link to protocol or registration | *Materials and Methods → PICO Model (“This review was not prospectively registered in PROSPERO or another public registry.”)* | Yes |
