# Supplementary material for: Liquid biopsies for the diagnosis of early-stage gastric cancer: A 5-year systematic review
Source: J Liq Biopsy. 2026 Apr 8;12:100465. doi: 10.1016/j.jlb.2026.100465 (PMC13092028; doi:10.1016/j.jlb.2026.100465)
Supplement: Multimedia component 2 [file mmc2.docx]

**Supplementary table 2: Full Search Strategy for each database**

| **Database** | **Platform / Provider** | **Search Interface** | **Search Strategy (Exact Query)** | **Limits / Filters Applied** | **Date Range** | **Date of Last Search** |
| --- | --- | --- | --- | --- | --- | --- |
| **PubMed** | NCBI | Native (PubMed.gov) | (("Stomach Neoplasms"[Mesh] OR "Gastric Cancer"[tiab] OR "Stomach Cancer"[tiab] OR "Gastric Neoplasm*"[tiab] OR "Stomach Neoplasm*"[tiab] OR "Gastric Oncology"[tiab] OR "Stomach Oncology"[tiab]))  AND  (("Liquid Biopsy"[Mesh] OR "liquid biops*"[tiab] OR "biomarker testing"[tiab] OR "non-surgical biops*"[tiab]))  AND  ("2020/01/01"[Date - Publication] : "2025/06/30"[Date - Publication]) | Humans, English | 2020–2025 | June 2025 |
| **Scopus** | Elsevier | Native (Scopus.com) | TITLE-ABS-KEY ("gastric cancer" OR "stomach cancer" OR "gastric neoplasm*" OR "stomach neoplasm*" OR "gastric oncology" OR "stomach oncology")  AND  TITLE-ABS-KEY ("liquid biops*" OR "biomarker testing" OR "non-surgical biops*")  AND  PUBYEAR > 2019  AND LANGUAGE (english)  AND DOCTYPE (ar) | Humans, English | 2020–2025 | June 2025 |
